# The Role of PI3K Inhibition in the Treatment of Breast Cancer, Alone or Combined With Immune Checkpoint Inhibitors

**DOI:** 10.3389/fmolb.2021.648663

**Published:** 2021-05-07

**Authors:** Zhizhu Zhang, Ann Richmond

**Affiliations:** ^1^Department of Biological Sciences, Vanderbilt University, Nashville, TN, United States; ^2^Department of Pharmacology, Vanderbilt University, Nashville, TN, United States; ^3^Department of Veterans Affairs, Tennessee Valley Healthcare System, Nashville, TN, United States

**Keywords:** PI3K inhibition, breast cancer, immune checkpoint inhibitors, MDSC, tumor immune microenvironment

## Abstract

Dysregulation of phosphoinositide 3-kinase (PI3K) signaling is highly implicated in tumorigenesis, disease progression, and the development of resistance to the current standard of care treatments in breast cancer patients. This review discusses the role of PI3K pathway in breast cancer and evaluates the clinical development of PI3K inhibitors in both early and metastatic breast cancer settings. Further, this review examines the evidence for the potential synergistic benefit for the combination treatment of PI3K inhibition and immunotherapy in breast cancer treatment.

## Introduction

Breast cancer is the most frequent cancer type in women worldwide (Bray et al., 2018). Breast cancer is generally divided into three subtypes, with targeted therapies determined by subtypes. Hormone receptor-positive (HR^+^)/HER2^–^ is the most common subtype and accounts for around 70% of breast cancer patients (Howlader et al., 2014). Treatment generally involves endocrine therapy (ET) (Waks and Winer, 2019). Recently, the approval of CDK4/6 inhibitors for use in combination with ET substantially improves survival outcomes (Shah et al., 2018). However, after resistance develops to hormone therapy, chemotherapy becomes the only standard treatment option (Cardoso et al., 2009); 15–20% of patients are diagnosed as HER2^+^ (Howlader et al., 2014). The standard-of-care treatment for HER2^+^ breast cancer incorporates HER2^–^ targeted antibody with chemotherapy (Romond et al., 2005; Waks and Winer, 2019). However, even after disease progression on therapy, continued administration of anti-HER2 and chemotherapy remains as the subsequent treatment (Olson et al., 2012). Accounting for 10–20% of newly diagnosed breast cancer cases, triple-negative breast cancer (TNBC) is characterized by the lack of hormone receptor expression and lack of HER2/NEU gene overexpression (Dent et al., 2007). TNBC demonstrates high heterogeneity in the mutational profile and shows the highest relapse risks (Dent et al., 2007). Due to a lack of targets, chemotherapy is currently the primary treatment for TNBC, but the clinical benefits are usually not durable due to frequently acquired resistance (Liedtke et al., 2008).

Upregulation of the phosphoinositide 3-kinase (PI3K) signaling pathway is commonly observed in breast cancer patients. It has been associated with breast cancer tumorigenesis, progression, and the development of resistance to hormone therapy and chemotherapy (Guerrero-Zotano et al., 2016; Drullinsky and Hurvitz, 2020). Therefore, it is essential to elucidate the mechanism of the PI3K signaling pathway in breast cancer and explore the potential of PI3K inhibitors in the treatment of different subtypes of breast cancer, alone or combined with other therapies in both early and metastatic settings. This review will provide an overview of the rationale for and development of PI3K inhibition and examine the potential of PI3K inhibitors to combine with immunotherapy in breast cancer treatment.

## Phosphoinositide 3-Kinase Signaling Pathway and Its Aberrant Activation in Breast Cancer

The PI3K signaling pathway is involved in many cellular processes, including glucose metabolism, cell growth, proliferation, and survival (Katso et al., 2001). PI3K is a family of lipid kinases. The Class I PI3Ks are the most commonly altered class in breast cancer and are composed of a heterodimer of a p85 regulatory subunit and a p110 catalytic subunit (Hiles et al., 1992). Upon receiving signals from receptor tyrosine kinases, PI3K catalyzes the conversion of phosphatidylinositol bisphosphate PI(4,5)P2 to phosphatidylinositol triphosphate PI(3,4,5)P3 (Schu et al., 1993). PIP3 recruits phosphoinositide-dependent kinase-1 (PDK1) and protein kinase B (AKT), thus promoting the activation of AKT by PDK1 phosphorylation (Alessi et al., 1997).

**FIGURE 1:**
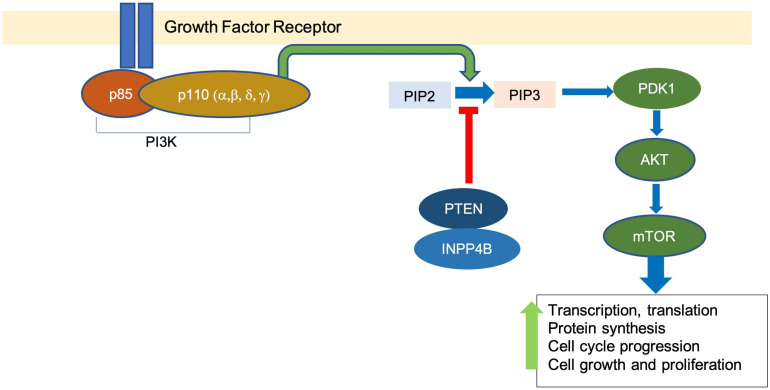
The overview of PI3K pathway. Binding of growth factor activates the tyrosine kinase receptor, which promotes the transformation of PIP2 to PIP3 by PI3K. This leads to a downstream signaling cascade that upregulates events associated with cell growth and proliferation.

AKT activation results in multiple downstream signaling cascades, including mammalian target of rapamycin (mTOR), which upregulates processes such as transcription and translation, protein synthesis, and cell cycle progression, among others (Sarbassov et al., 2005). Negative regulators of the pathway include phosphate and tensin homolog (PTEN) and inositol polyphosphate 4-phosphatase (INPP4B), which dephosphorylate PIP3 and convert it back to PIP2 (Maehama and Dixon, 1998; Gewinner et al., 2009; Figure 1).

Several genetic aberrations associated with genes that encode for proteins in the PI3K signaling pathway have been identified in breast cancer. Mutations in the p110α subunit (PIK3CA) have been found in around 40% of HR^+^/HER2^–^ or HER2^+^ advanced breast cancer tumors and around 9% of TNBC tumors (Cancer Genome Atlas Network, 2012; Guerrero-Zotano et al., 2016). Mutations in other subunits of p110 are much rarer. In TNBC tumors, inactivating mutations mutations, loss of PTEN, or heterozygous deletion of INPP4B are the more frequently observed PI3K pathway-related genetic alterations (Cancer Genome Atlas Network, 2012). Mice bearing TNBC tumors engineered with an INPP4B deficiency showed increased sensitivity to PI3K inhibition (Liu et al., 2020).

Therapy resistance across breast cancer subtypes can result from PI3K overactivation. For HR^+^ breast cancer, ET serves as the first-line treatment. However, resistance to ET commonly arises. Evidence suggests that antiestrogen resistance might be conferred by the PI3K pathway (André et al., 2019). Increased PI3K pathway signaling is associated with the downregulation of endocrine receptor expression, which could lead to insensitivity to hormone therapies (Creighton et al., 2010). Treatment with the dual PI3K/mTOR inhibitor BEZ235 in the long-term estrogen-deprived, hormone-resistant cell line resulted in restored sensitivity to tamoxifen, induced apoptosis, and increased ER levels (Creighton et al., 2010). In HER2^+^ cancers, resistance to the standard-of-care anti-HER2 antibody trastuzumab has also been associated with the constitutive activation of the PI3K pathway (Pohlmann et al., 2009). In trastuzumab-resistant breast cancer cell lines due to the PTEN deficiency, inhibition of PI3K pathway restores sensitivity to trastuzumab and significantly inhibits cell growth (Junttila et al., 2009). In TNBC, dysregulation in the PI3K pathway has been associated with chemotherapy resistance (Drullinsky and Hurvitz, 2020).

## The Clinical Development of PI3K Inhibitors: Efficacy, Resistance, and Toxicity

Multiple PI3K inhibitors have been developed and evaluated in various stages of clinical trials. PI3K inhibitors can be divided into pan-PI3K inhibitors, isoform-specific PI3K inhibitors, and dual PI3K/mTOR inhibitors.

Pan-PI3K inhibitors target multiple isoforms of the Class I PI3K. Buparlisib targets all four isoforms (α, β, δ, γ) and, when combined with fulvestrant, showed only a moderate improvement in the median progression-free survival (PFS) for about 1.9 months compared with the placebo and fulvestrant group in HR^+^/HER2^–^ advanced breast cancer patients (Baselga et al., 2017). However, broad targeting of multiple forms of PI3K isozymes leads to a high rate of severe adverse events (AEs) that mandate dose reduction, interruptions, and discontinuation, thus discouraging further investigation (Baselga et al., 2017). Selective inhibitors for a combination of a few isoforms have been developed against solid tumor and hematological malignancies. Copanlisib targets PI3Kα and PI3Kδ isoforms and has been approved as a treatment for relapsed follicular lymphoma (FL) (Dreyling et al., 2017). Phase Ib/II studies are ongoing in evaluating the efficacy of Copanlisib in pretreated recurrent or metastatic HER2^+^ BC in combination with trastuzumab (Keegan et al., 2018). Taselisib, a β-isoform-sparing pan-PI3K inhibitor, when combined with fulvestrant in the treatment of PIK3CA-mutated ER^+^/HER2^–^ locally advanced or metastatic BC, showed only a moderate 2-month improvement in median PFS (7.4 vs. 5.4) over the placebo arm (Baselga et al., 2018). Duvelisib targets PI3Kδ and PI3Kγ, which are isoforms highly expressed in lymphoid cells and implicated in malignant B- and T-cell proliferation and survival (Flinn et al., 2018). Duvelisib has been approved for the treatment of chronic lymphocytic leukemia (CLL) and FL (Flinn et al., 2018). However, PI3Kδ or PI3Kγ is not commonly mutated in breast cancer, and a direct stimulatory effect of PI3Kδ or PI3Kγ in breast cancer tumor growth is not obvious. The potential indirect benefit of targeting PI3Kδ or PI3Kγ in breast cancer for enhancing anti-tumor immunity will be discussed in the following section.

Isoform-specific PI3K inhibitors target only one or a few selected isoforms among the four. Alpelisib specifically inhibits p110α, the major mutated isoform in the PI3K pathway in breast cancer, as discussed above. In the SOLAR-1 trial, a combination of alpelisib with fulvestrant showed a 5.3 month improvement in mPFS compared with the placebo-fulvestrant group in patients with PIK3CA mutation (André et al., 2019). The rates of AEs are also lower than pan-PI3K inhibitors, indicating a more tolerable toxicity profile that allows for more prolonged and uninterrupted dosing schedule and higher doses. The discontinuation rate is 25.0 vs. 4.2% in alpelisib vs. placebo, compared with 39 vs. 5% in the buparlisib study (Baselga et al., 2017; André et al., 2019). This result prompted alpelisib as the first approved PI3K inhibitor for HR^+^/HER2^–^ PIK3CA-mutated advanced/metastatic BC patients after progression on ET. However, alpelisib with letrozole for the neoadjuvant treatment of HR^+^/HER2^–^ early breast cancer patients reported no improvement in ORR regardless of whether the patients have PIK3CA mutation (Mayer et al., 2019). Further evaluation of alpelisib in TNBC and HER2^+^ settings might provide encouraging results.

Although normal or PTEN-WT breast tumors do not depend on p110 β isoform activity in PI3K signaling, cancer cells with PTEN deficiency require p110β activity to sustain PI3K signaling and are sensitized to PI3Kβ-specific inhibition (Torbett et al., 2008). This suggests that while PI3Kα inhibition may be optimally tailored toward PIK3CA-mutated breast tumor types, PI3Kβ inhibition may be exploited for PTEN-deficient breast tumors, notably for TNBC. Research in the area of PI3Kβ inhibition for breast cancer is limited. A recent preclinical study evaluated the effect of AZD8186, a selective PI3Kβ/δ inhibitor, on mice injected with PTEN-deficient TNBC tumors, but demonstrated mediocre anti-tumor effect as a single agent or combined with paclitaxel (Owusu-Brackett et al., 2020). The lack of efficacy could be due to the feedback loop between PI3Kα and PI3Kβ, leading to a reactivation of the signaling that became inhibited with dual inhibition of the isoforms in prostate cancer (Schwartz et al., 2015). Other examples of isoform-specific PI3K inhibitors include idelalisib, an inhibitor specific for p110δ, approved for CHL treatment due to a significantly improved survival profile (Furman et al., 2014).

Dual PI3K/mTOR inhibitors compete for the ATP-binding cleft of PI3K and mTOR kinases (Guerrero-Zotano et al., 2016). However, phase I/II clinical trials for dual PI3K/mTOR inhibitors dactolisib (BEZ235), apitolisib (GDC-0980), and gedatolisib (PF-05212384) showed a relatively poor safety profile that results in low tolerable dosage and modest anti-tumor responses (Dolly et al., 2016; Wainberg et al., 2017; Wise-Draper et al., 2017). Several clinical studies are ongoing to explore their efficacy in breast cancer. Gedatolisib is being tested in treatment for metastatic TNBC and ER^+^ patients (Forero-Torres et al., 2018; Radovich et al., 2018).

The limited efficacy of PI3K inhibitors could be attributed to the complex feedback loops in the PI3K/AKT/mTOR signaling network and its crosstalk with other signaling pathways. This results in subsequent reactivation of PI3K or parallel pathway that confers resistance and reduces the anti-tumor effects substantially. In PTEN-deficient tumor cells, resistance to a pan-PI3K inhibitor was conferred by over-activation of p110-beta (Nakanishi et al., 2016). The PI3K pathway engages in crosstalk with the RAS-RAF-MEK-ERK pathway at multiple nodes of interaction (Castellano and Downward, 2011). In HER2 amplified breast cancer cells, although PI3K inhibitors successfully suppress AKT activation downstream, there is enhanced activation of HER family receptors and a compensatory activation of the ERK signaling (Serra et al., 2011). Combined administration of anti-HER2 monoclonal antibodies and MEK inhibitors could antagonize the proliferative effect resulting from ERK dependency upon PI3K inhibition (Serra et al., 2011). A study using murine cancer models showed a combination of MEK and PI3K/mTOR inhibitor resulted in significant tumor regression and survival advantages in both basal-like and HER2^+^ subtype models (Roberts et al., 2012). However, their data suggested that resistance to the triplet inhibition will still develop within months. Additionally, combined treatment could lead to a high toxicity profile, and the need for treatment breaks; as a result, significant weight loss in mice induces tumor regrowth (Roberts et al., 2012). Further research that elucidates the possible mechanism of PI3K inhibition, intrinsic and acquired resistance, is essential to enhance the durability of clinical benefit with targeted combination treatment tailored to specific subtypes of breast cancer and the mutational status of the patients.

Phosphoinositide 3-kinase inhibitors could lead to many AEs. Hyperglycemia is one of the most observed AEs across clinical trials (Drullinsky and Hurvitz, 2020). These AEs are related to the normal function of p110α in promoting insulin signaling, resulting in the breakdown of glycogen, minimal glucose uptake, and high glucose level in the circulatory system (Hopkins et al., 2018). Considered as an on-target result of PI3K inhibition, reduced insulin also impedes tumor cell proliferation by impairing their uptake of glucose (Hopkins et al., 2018). Gastrointestinal AEs are also commonly observed with PI3K inhibition. Severe diarrhea associated with PI3Kδ isoform inhibition is due to the impairment of colon macrophage functionality and pro-inflammatory response (Uno et al., 2010). This again justifies selective targeting of PI3K isoforms based on the observed mutational status of the patients to reduce AEs.

Overall, despite the importance of the PI3K pathway in breast cancer and therapy resistance, many PI3K inhibitors used as monotherapy reported limited efficacy and high toxicity profile in current clinical trials across breast cancer subtypes. This necessitates the examination of PI3K inhibition in combination with other types of therapies to potentially maximize anti-tumor effects.

## Immunotherapy in Breast Cancer

Immunotherapies based on the use of immune checkpoint inhibitors (ICI) target T-cell co-inhibitory signalings, namely, CTLA-4 and PD-1/PD-L1, and hence relieve their suppression of anti-tumor T-cell activity and prevent tumor immune evasion (Hargadon et al., 2018). ICI treatment exhibits significantly improved survival rate and inhibition of tumor growth across the treatment of various types of tumors, leading to FDA approval in many cancer indications (Table 1). However, single-agent treatment using monoclonal antibodies against PD-1 or PD-L1 generally reported mediocre efficacy in breast cancer patients since most breast cancer tumors have long been classified as immunologically “cold.” “Cold” tumors are highly unresponsive to immunotherapy treatment (Szekely et al., 2018).

**TABLE 1:** The FDA-approved immune checkpoint inhibitors and their approved indications.

Checkpoint target	FDA-approved drug	Approved indication(s)
Immune cytotoxic	Ipilimumab	Melanoma
T-lymphocyte		*Advanced renal cell carcinoma
antigen-4 (CTLA-4)		*MSI-H/dMMR metastatic colorectal cancer
		*Hepatocellular carcinoma
		*NSCLC
		*Malignant pleural mesothelioma
Programmed	Pembrolizumab	NSCLC
Death-1 (PD-1)		Squamous cell head and neck cancer
		Melanoma
		Merkel cell carcinoma
		Hepatocellular carcinoma
		MSI-H/dMMR cancer
		Cervical cancer
		Gastric carcinoma
		Classical Hodgkin’s lymphoma
		PMBCL
		Urothelial bladder cancer
		Non-muscle invasive bladder cancer
		Advanced renal cell carcinoma
		Esophageal squamous cell carcinoma
		Cutaneous squamous cell carcinoma
	Nivolumab	Small cell lung cancer
		NSCLC
		Squamous cell head and neck cancer
		Melanoma
		Hepatocellular carcinoma
		Advanced renal cell carcinoma
		Urothelial cancer
		Esophageal squamous cell carcinoma
		Malignant pleural mesothelioma
		Classical Hodgkin lymphoma
		MSI-H/dMMR metastatic colorectal cancer
	Cemiplimab	Cutaneous squamous cell carcinoma
Programmed	Atzolizumab	Small cell lung cancer
Death-Ligand 1		NSCLC
(PD-L1)		TNBC
		Urothelial cancer
		Hepatocellular carcinoma
		Melanoma
	Durvalumab	NSCLC
		Small cell lung cancer
		Urothelial carcinoma
	Avelumab	Merkel cell carcinoma
		Urothelial cancer
		Renal cell carcinoma

*The approved use of ipilimumab is in combination with nivolumab. NSCLC, non-small cell lung cancer; PMBCL, primary mediastinal large B-cell lymphoma; MSI-H/dMMR, microsatellite instability high/deficient mismatch repair.

Triple-negative breast cancer is considered as the most immunogenic subtype of breast cancer, with a higher lymphocyte infiltration rate than HER2^+^ or HR^+^ tumors and thus regarded as a promising target for immunotherapies (Szekely et al., 2018). PD-L1 is a commonly overexpressed biomarker in TNBC (Mittendorf et al., 2014). TNBCs showed responsiveness to immunotherapies combined with chemotherapies in clinical trials, leading to the approval of both atezolizumab and pembrolizumab by the FDA to use in combination with chemotherapy for PD-L1 positive, unresectable, locally advanced, or metastatic TNBC (Cortes et al., 2020; Narayan et al., 2020). Pembrolizumab plus chemotherapy has demonstrated a 4.1 month improvement in median PFS over the placebo arm (9.7 vs. 5.6) (Cortes et al., 2020).

Based on tumor-infiltrating lymphocyte count and PD-L1 expression, primary breast cancer tumors show higher immunogenicity than the metastatic tumor samples (Szekely et al., 2018). Therefore, neoadjuvant and adjuvant immunotherapy targeting early stage breast cancer has been an area of increasing interest. In the phase 2 trial I-SPY2, pembrolizumab with neoadjuvant chemotherapy showed a 17% improvement (30 vs. 13%) in the estimated pathologic complete response (pCR) in HR^+^/HER2^–^ patients and a 38% improvement (60 vs. 22%) in the estimated pCR in TNBC patients as compared to the placebo (Nanda et al., 2020).

## The Rationale for PI3K Inhibition Combination Approach With Immunotherapy

With the recent breakthroughs in both PI3K inhibitor alpelisib and immune checkpoint blockade (ICB) treatment in breast cancer, the potential for PI3K inhibition in combination with PD-1/PD-L1 blockade merits evaluation. Increasing evidence suggests that in addition to the direct proliferative effects on tumor cells, the PI3K-AKT-mTOR pathway is involved in creating an immunosuppressive tumor microenvironment. Okkenhaug et al. (2016) and O’Donnell et al. (2018) provided extensive reviews on the recent findings of the immunomodulatory roles of PI3K pathway on the tumor microenvironment, including the enhanced expression of PD-L1, recruitment, and differentiation of myeloid-derived suppressor cells (MDSCs) and Tregs into the tumor, and secretion of suppressive cytokines to impair stimulation of macrophages and dendritic cells and the migration, expansion, functionality, and memory development of T cells (Okkenhaug et al., 2016; O’Donnell et al., 2018). These authors hypothesized that inhibition of the PI3K pathway may further boost T-cell-mediated tumor killing when exploited for therapeutic combination with immunotherapy. However, whether the stimulatory effects of PI3K inhibition on anti-tumor immunity translate to breast cancer and sensitize breast tumors for immune checkpoint inhibition is not specifically discussed. This review will summarize the research results and clinical trials in the setting of breast cancer and will suggest a future direction to explore the synergy for combining PI3K inhibitors and ICI in breast cancer.

Phosphate and tensin (PTEN) homolog loss has been identified as one mechanism that induces PD-L1 expression in TNBC via transcriptional regulation, while inhibition of the PI3K pathway with the administration of an AKT inhibitor leads to decreased PD-L1 expression (Mittendorf et al., 2014). This establishes the connection between PTEN, the PI3K pathway, and the regulation of PD-L1 expression. The increased PD-L1 expression induced by PTEN loss leads to reduced proliferation and survival of T cells, inhibiting effective antitumor adaptive immunity (Mittendorf et al., 2014). Peng et al. showed that in the murine melanoma model, the immune resistance conferred by PTEN loss, including the reduction in T-cell infiltration and expansion, can be reversed by selective inhibition of PI3Kβ in combination with ICI treatment (anti-PD-1 and anti-CTLA-4). The combination treatment significantly reduces tumor progression, in contrast to the limited efficacy of treatment with every single agent (Peng et al., 2016). As suggested in Pascual and Turner (2019), the same synergistic potential could be exploited in PTEN-deficient TNBC.

p110δ and p110γ are mostly expressed in leukocytes and thus have been targeted for hematologic malignancies. Increasing evidence points to their role in immunosuppressive mechanisms on T cell and myeloid cell activity, suggesting the clinical potential for PI3Kδ and PI3Kγ inhibitors to enhance the anti-tumor sensitivity of the immune system across cancer types, including preclinical breast tumor models. The immunosuppressive effect of p110γ on the tumor microenvironment is demonstrated by its role in the recruitment of MDSCs into the tumor tissues. MDSCs are a heterogeneous population of immature myeloid cells that are known to induce suppression of the antitumor function, proliferation, and migration of immune cells, including T cell and NK cells, and promote tumor angiogenesis and metastases (Umansky et al., 2016). Selective inhibition or genetic knockout of p110γ significantly reduces α4β1 integrin-mediated trafficking and adhesion of MDSCs to the tumor, leading to the suppression of spontaneous breast carcinoma growth (Schmid et al., 2011). PI3Kγ-specific inhibition is also shown to polarize the myeloid cells to the more immune-stimulatory M1-like phenotype from the immunosuppressive M2-like phenotype in tumor models including 4T1 breast cancer, which correlates with higher CD8/Treg ratio from increased CD8^+^ T-cell infiltration (De Henau et al., 2016; Kaneda et al., 2016). A similar observation of enhanced CD8^+^ T-cell infiltration is observed in another study with PI3Kγ knockout mice injected with MMTV-PyMT tumor cells, which also correlates with slower growth of the tumor and reduced metastasis to lung (Sai et al., 2017).

The subversion of tumor-infiltrating myeloid recruitment by PI3Kγ inhibitor has the potential to enhance TNBC sensitivity to ICI. Kim et al. categorized TNBC models into neutrophil-enriched and macrophage-enriched subtypes. While the macrophage-enriched subtype (MES) exhibits increased CD8^+^ T-cell infiltration and activity upon exposure to ICI, neutrophil-enriched subtypes (NES) of TNBC models respond minimally to ICI treatment due to the highly immunosuppressive microenvironment created by granulocytic MDSC accumulation (Kim et al., 2019). Acquired resistance to ICI treatment by the previously responsive MES tumor is also associated with increased accumulation of gMDSCs (Kim et al., 2019). Granulocytic MDSCs are similar to neutrophils in morphological and phenotypical ways and constituted the majority of the MDSC population in most types of cancer (Gabrilovich, 2017). The Gene Set Enrichment Analysis (GSEA) associates the high tumor-associated neutrophil level to PI3K-AKT-mTOR pathway, which aligns with the previous finding of the group showing that mTOR signaling stimulates MDSC accumulation in the mammary tumor (Welte et al., 2016). The PI3K pathway has also been associated with immunosuppressive, neutrophil-recruiting cytokines and chemokines such as CXCL1 and CXCL-8, implicating the mechanism for MDSCs recruitment (Kim et al., 2019). Thus, targeting PI3K-mediated MDSC infiltration may sensitize intrinsic and acquired resistant breast tumors for ICI treatment. The preclinical combination of PI3Kγ inhibition and ICI showed promising outcomes: co-administration of PI3Kγ inhibitor with either anti-PD1 or anti-CTLA-4 significantly inhibits 4T1 tumor growth compared with ICI treatment alone (De Henau et al., 2016). Whether other components of the PI3K pathway besides PI3Kγ also modulate the ICI-resistance from MDSC infiltration in TNBC merits more investigation.

Inactivation of p110δ by genetic ablation or selective inhibition has been shown to reduce breast tumor growth and metastasis in mouse 4T1 model and other models of solid cancer by impairment of Treg function, including suppression in their secretion of interleukin (IL)-10 (Ali et al., 2014). Significantly, data from p110δ-inactivated mice suggest effective anti-tumor memory formation, with significant suppression of relapse from the surgical removal of breast tumor compared to wild type (Ali et al., 2014). This result is consistent with a study that revealed enhanced persistence of the CD8^+^ memory T cells upon PI3Kδ inhibition in a melanoma model (Abu Eid et al., 2017).

**FIGURE 2:**
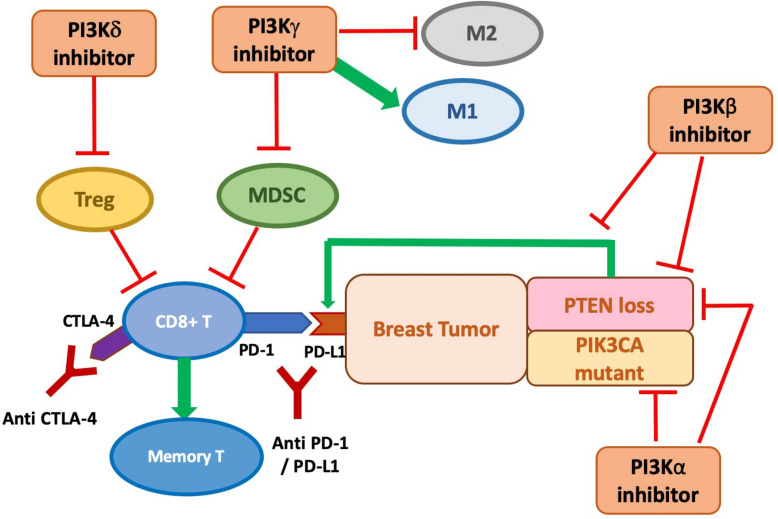
The anti-tumor role of PI3K inhibitors on breast cancer with direct inhibition on cancer cell proliferation and indirect stimulation of the immune response, and potential synergy with immune checkpoint inhibition. PI3Kδ inhibitor is shown to suppress Treg function. PI3Kγ inhibitor may prevent MDSC infiltration and accumulation and polarize macrophages to the M1 phenotypes. PI3Kβ inhibition antagonizes the p110β activity required for PTEN-null tumor and targets the increased PD-L1 expression on PTEN-null tumor. PI3Kα inhibition is demonstrated to be effective at preventing tumor cell growth, especially in PIK3CA mutants, but might also be promising in PTEN-null tumor to prevent the PI3Kβ/α feedback loop.

The role and importance of each PI3K isoform in shaping the anti-tumor immune response in breast tumors still need further characterization to devise the optimal selective inhibition scheme for combination with ICI treatment. Given the complex direct and indirect anti-tumor role of different PI3K isoforms, the combination of a pan-PI3K inhibitor with ICI might also provide clinical advantages. Moreover, PTEN loss has been identified as one mechanism that induces PD-L1 expression in TNBC via transcriptional regulation. Simultaneously, inhibition of the PI3K pathway with the administration of an AKT inhibitor leads to decreased PD-L1 expression (Mittendorf et al., 2014). This establishes the connection between PTEN, the PI3K pathway, and the regulation of PD-L1 expression. The increased PD-L1 expression induced by PTEN loss leads to reduced proliferation and survival of T cells, inhibiting effective anti-tumor adaptive immunity (Mittendorf et al., 2014). Thus, there is a synergistic potential of anti-PD-L1 immunotherapy and targeted inhibition of the PI3K-AKT-mTOR pathway (Figure 2).

Preclinical studies with this combination scheme in breast cancer are still limited, but some show promising results. One study found that despite limited efficacy of anti-PD1 monotherapy in mice model bearing PyMT tumor, the co-treatment of a pan-PI3K inhibitor with anti-PD1 antibody leads to a significantly smaller mean tumor volume than with both agents used alone (Sai et al., 2017). Another preclinical study performed a quadruple combination using PI3Kα, CDK4/6, PD-1, and CTLA-4 inhibition. It showed highly durable tumor regression in the TNBC model, with a significant increase in granzyme B positive CD8^+^ and CD4^+^ T cells (Teo et al., 2017).

Several phase I/II clinical trials are ongoing to assess the combination of ICI with inhibitors of the PI3K/AKT/mTOR pathway, mostly in lymphoma and leukemia (Table 2). There are a few that look at this combination therapy in TNBC, including two trials that evaluate one PI3Kγ inhibitor: one with atezolizumab (NCT03961698) and one with nivolumab (NCT02637531). Another trial combines PI3Kδ inhibition with pembrolizumab (NCT02646748). Other clinical studies are in progress to investigate the potential synergistic benefit of AKT or mTOR inhibitors with ICIs (NCT03742102; NCT03961698; NCT02890069).

**TABLE 2:** Ongoing clinical trials combining immune checkpoint inhibitors with inhibitors of the PI3K/AKT/mTOR pathway.

Combination	Drug names	Indications	Phase	Clinical trial identifier
Anti-PD1 + PI3K inhibitor	Pembrolizumab + Copanlisib	Relapsed or refractory NK and T-cell non-Hodgkin lymphoma	I/II	NCT02535247
	Pembrolizumab + Duvelisib	Recurrent or metastatic (R/M) head and neck squamous cell carcinoma (HNSCC)	I/II	NCT04193293
	Pembrolizumab + Idealisib	NSCLC, relapsed or refractory chronic lymphocytic leukemia	Ib/II	NCT03257722, NCT02332980
	Pembrolizumab + PI3K-beta inhibitor GSK2636771	Metastatic melanoma and PTEN loss	I//II	NCT03131908
	Nivolumab + PI3K-gamma inhibitor IPI-549	Advanced urothelial carcinoma	II	NCT03980041
	Pembrolizumab + PI3K-delta inhibitor Umbralisib	Relapsed or refractory chronic lymphocytic leukemia	II	NCT03776864
	Nivolumab + Duvelisib	Richter syndrome or transformed follicular lymphoma	I	NCT03892044
	Nivolumab + Eganelisib (PI3Kγ)	Advanced solid tumors, NSCLC, melanoma, triple negative breast cancer, adrenocortical carcinoma, mesothelioma, high-circulating myeloid-derived suppressor cells	1/1b	NCT02637531
	Pembrolizumab + INCB050465 (PI3Kδ)	Colorectal cancer, endometrial cancer, melanoma, head and neck cancer, lung cancer, MMR-deficient tumors, breast cancer, pancreatic cancer, renal cell carcinoma, solid tumors, urothelial cancer	1	NCT02646748
Anti PD-L1 + AKT inhibitor	Durvalumab + Capivasertib + Paclitaxel	Metastatic triple-negative breast cancer	I/II	NCT03742102
Anti PD-L1 + PI3K inhibitor	Atezolizumab + PI3K-gamma inhibitor IPI-549	Triple-negative breast cancer, renal cell carcinoma	II	NCT03961698
Anti PD-L1 + mTOR inhibitor	Durvalumab + Sirolimus	Stage I-IIIA non-small cell lung cancer	I	NCT04348292
Anti-PD1 + mTOR inhibitor	PDR001 + Everolimus	Colorectal cancer, non-small cell lung carcinoma, triple negative breast cancer	Ib	NCT02890069

**Clinical trial information from clinicaltrials.gov.**

## Discussion

With the approval of the alpha selective PI3K inhibitor alpelisib in HR^+^/HER2^–^ PIK3CA-mutated advanced/metastatic breast cancer patients after progression on ET, several clinical trials evaluate its efficacy in advanced TNBC, and HER2^+^ subtypes are ongoing (NCT04208178; NCT04216472). The success of SOLAR-1 demonstrates that stratification of patients by PIK3CA mutation is promising to identify the patient population that is more likely to respond maximally to PI3K alpha isoform inhibition. However, besides PIK3CA mutation, other potential biomarkers for predicting the efficacy of PI3K inhibition require further evaluation. PIK3CA mutation is not as common in the highly heterogenous TNBC patients and might exclude many patients from receiving PI3K inhibition treatment. Additionally, although activating PIK3CA mutation is generally associated with greater therapeutic response, some patients without this mutation benefited significantly from PI3K alpha isoform inhibition, while others with PIK3CA mutation exhibit minimal benefit (André et al., 2019). Thus, identifying more reliable biomarkers for patient selection would be critical for optimizing response to PI3K inhibition across breast cancer subtypes. Inevitably, this is dependent on the elucidation of the details of aberrant PI3K signaling in the different breast cancer subtypes and resistance mechanisms in response to PI3K inhibition and to previous or concurrent treatment.

PD-L1 expression has been the inclusion criteria for using anti-PD-1 or anti-PD-L1 immunotherapy drugs in TNBC patients. Another emerging biomarker for the prediction of patient response to ICIs independent of PD-L1 expression is the tumor mutation burden (TMB), which measures the total number of somatic mutations in a tumor genome (Chan et al., 2019). In melanoma and lung cancer, TMB has been suggested to associate with higher lymphocytic infiltration and enhanced immunogenicity with responsiveness to ICIs (Chan et al., 2019). However, the role of TMB and its relation to PD-L1 expression in BC has not yet been well elucidated (Ravaioli et al., 2020). No study has yet explored the biomarkers that could predict maximized effectiveness and reduced AEs for the combination treatment of immunotherapy and PI3K inhibition. Considering the role of PI3K signaling in the recruitment of MDSCs, regulatory T cells, and expression of PD-L1, appropriate biomarkers might be mutations in the PI3K pathway that are specifically involved in creating an immunosuppressive tumor microenvironment.

Although breast cancer subtypes other than TNBC have been considered “cold tumor” unresponsive to immunotherapy, in light of the greater immunogenicity of the primary tumor and improved PCR in HR^+^/HER2^–^ early breast cancer patients with anti-PD-1 drugs in the I-SPY2 trial, this suggests the potential to extend immunotherapy beyond TNBC in early breast cancer treatment, and combination paradigm with PI3K inhibition could further transform the immunosuppressive environment and enhance T-cell infiltration. Given the toxicity of both PI3K inhibition and immunotherapy, the tolerability of dual combination or the addition of more types of therapies in the treatment regimens warrants careful investigation. With the potential mechanism of synergy, combination treatment might allow decreased toxicity by achieving greater efficacy with lower dosing needs.

Overall, the PI3K pathway is highly implicated in the tumorigenesis, progression, and intrinsic and acquired resistance to current anti-tumor treatment in breast cancers. Although several PI3K inhibitors have been tested, except for alpelisib, many currently demonstrated disappointing efficacy with intolerable toxicity in breast cancer patients. Also, only recently has immunotherapy been approved as an option for TNBC patients. With the possible synergistic benefit, there is considerable potential for a combination treatment for these two newly approved options for breast cancer: PI3K inhibition with immunotherapy. Further understanding of the PI3K signaling and crosstalk with related pathways, its role in activating or suppressing the tumor-infiltrating lymphocytes, and better patient stratification and selection strategies will be essential patient response in the clinical development of PI3K inhibitors and their possible combination approach with immunotherapies.

## Author Contributions

ZZ and AR wrote the manuscript. Both authors contributed to the article and approved the submitted version.

## Conflict of Interest

The authors declare that the research was conducted in the absence of any commercial or financial relationships that could be construed as a potential conflict of interest.
